# Software Testing of eHealth Interventions: Existing Practices and the Future of an Iterative Strategy

**DOI:** 10.2196/56585

**Published:** 2024-07-19

**Authors:** Oyinda Obigbesan, Kristen Graham, Karen M Benzies

**Affiliations:** 1 Cumming School of Medicine University of Calgary Calgary, AB Canada; 2 Haskayne School of Business University of Calgary Calgary, AB Canada; 3 Faculty of Nursing University of Calgary Calgary, AB Canada

**Keywords:** eHealth, health system, digital health, mHealth, mobile health, app, software testing, alpha testing, beta testing, usability testing, agile development, health applications, software, usability, literature review, narrative review, testing, ICT, information and communication technology, reliability, safety

## Abstract

eHealth interventions are becoming a part of standard care, with software solutions increasingly created for patients and health care providers. Testing of eHealth software is important to ensure that the software realizes its goals. Software testing, which is comprised of alpha and beta testing, is critical to establish the effectiveness and usability of the software. In this viewpoint, we explore existing practices for testing software in health care settings. We scanned the literature using search terms related to eHealth software testing (eg, “health alpha testing,” “eHealth testing,” and “health app usability”) to identify practices for testing eHealth software. We could not identify a single standard framework for software testing in health care settings; some articles reported frameworks, while others reported none. In addition, some authors misidentified alpha testing as beta testing and vice versa. There were several different objectives (ie, testing for safety, reliability, or usability) and methods of testing (eg, questionnaires, interviews) reported. Implementation of an iterative strategy in testing can introduce flexible and rapid changes when developing eHealth software. Further investigation into the best approach for software testing in health care settings would aid the development of effective and useful eHealth software, particularly for novice eHealth software developers.

## Introduction

eHealth interventions are becoming a part of standard care, with mobile apps or software solutions being created for patients and health care providers. For example, we designed a digital audit software for a new model of care called Merge (which is better known in research and development as Alberta Family Integrated Care) that improves outcomes by integrating families of newborns with critical illness into the neonatal intensive care unit (NICU) team [1]. Merge was adopted in all 14 NICUs in Alberta, Canada, through a process that involves quarterly fidelity audits, which are central to implementation success and sustainability. We replaced the manual and labor-intensive audits with this fidelity audit software to increase efficiency and reduce personnel costs.

Appropriate software testing is important to ensure that an eHealth intervention realizes its goals, which may include improving access, efficiency, and quality of care [2]. Software testing, which includes alpha and beta testing, is critical to establish the effectiveness and usability of an eHealth software. In this viewpoint, we define alpha testing in accordance with the Guide to the Software Engineering Body of Knowledge [3], as internal software testing, which occurs first and is often performed within the development team [4]. Subsequently, beta testing is performed, which is external and involves a larger testing sample representative of the end-user population [3,4]. When we set out to test the Merge fidelity audit software, we wanted to identify software testing practices for novice eHealth software developers. Thus, we performed an exploratory scan of the literature to understand and identify existing methods for testing software in health care settings.

For our exploratory scan, we used Google Scholar and search terms related to eHealth software testing (eg, “health alpha testing,” “eHealth testing,” and “health app usability”). We included research articles that described their process of either alpha or beta testing and were either patient- or health care provider–facing. We excluded articles if they were nonpeer-reviewed literature published before 2015 from a non-Organisation for Economic Co-operation and Development member country because we were interested in recent practices conducted in similar health care contexts. We identified 34 articles that met our inclusion criteria; among these, we selected 7 articles for this viewpoint about software testing practices (Multimedia Appendix 1), including 5 primary research articles [5-9], 1 review article [10], and 1 framework [11].

## Lack of a Standardized Testing Framework

There was not a single standardized framework for software testing that was used across the 5 primary research articles [5-9]; 2 articles [7,8] cited two different frameworks, while the other 3 articles did not cite any. Fishbein et al [8] used Darlow and Wen’s [12] best practices to guide the development of their mobile health (mHealth) intervention. The practices relevant for software testing included using mixed methods (eg, questionnaires, semistructured interviews), engaging stakeholders (eg, software designers, subject matter experts, health professionals, patients), and publishing the results of testing to facilitate learning from successes and difficulties [12]. Cho et al [7] used the 3-level stratified view of health IT usability evaluation [13]. Levels of testing, as defined by this framework, consisted of (1) a user-centered design, which incorporated users’ needs in the development phase; (2) a usability evaluation in a laboratory setting, where testing occurred in a regulated environment; and (3) usability in a real-world setting, which assessed user experience in practical applications [7,13]. In Cho et al [7], levels 2 and 3 most closely correspond to beta testing, as testers are representative of the end-user population [7]. While Darlow and Wen’s [12] best practices provide guidelines regarding what the testing should include, the stratified view of health IT usability evaluation [13] provides more specific guidance on how to conduct testing and what each phase may entail. However, both frameworks involve multiple methods of testing to gain comprehensive user feedback and aim to create standardization in testing to ensure the rigor of eHealth interventions. The lack of standardization in software testing raises issues concerning consistency in quality assurance, which has the potential to compromise patient safety. Additionally, the lack of clear guidelines for testing can lead to inefficiencies in the allocation of the development team’s time and resources.

## Objectives and Methods of Testing

Although few articles used a specific framework for testing, there were some similarities in the general process among the 5 primary research articles[5-9] (Multimedia Appendix 1). For both alpha and beta testing, this involved defining the objectives of testing, selecting testers and methods of evaluation, collecting and analyzing data, and refining the software.

The desired result of testing was the evaluation of different aspects of the eHealth software. Only 2 articles [5,6] clearly stated their objectives for testing. Ahonen et al [5] planned to identify content acceptability, feasibility, and technical issues during alpha testing of their eLearning intervention. For their mHealth intervention, Athilingam et al [6] aimed to evaluate design and functionality during alpha testing, followed by evaluations of helpfulness, usability, and design during beta testing. In addition to these objectives, software testing was used to evaluate safety, reliability, effectiveness, satisfaction, and accessibility, and to determine if the software fulfilled its intended purpose [14]. While the objectives of testing may overlap between alpha and beta testing, alpha testing may have a greater focus on software-specific objectives (eg, safety and bug fixes) and beta testing may focus on user experience–related objectives (eg, design and satisfaction) [4].

Selecting testers was an important testing step mentioned in all articles that described testing; however, the distinction between alpha and beta testing was often obscured at this stage. Several authors [5,6] stated that they were conducting alpha testing, yet the testing sample included members of the end-user population, which is more appropriately aligned with the definition for beta testing. For example, Athilingam et al [6] described alpha testing of their mobile app for patients with heart failure, but the testing sample consisted of the target end users (ie, people with a history of heart failure). Additionally, there was no clear justification for the size of the testing sample, which ranged from 2 to 76 testers [5-9]. In a scoping review, the sample size for software testing was found to vary according to the method of testing, with studies based on qualitative methods having fewer testers than those based on quantitative methods [10]. Most studies opted for a larger number of testers and tested their software only once. Hoffiman et al [9] had only 2 alpha testers who tested their Public Open Space Tool software on 55 different green spaces. The feasibility of this strategy depended on the same tester generating new insights during each round of alpha testing.

The most common methods of testing were questionnaires, “think-aloud” techniques, interviews, and focus groups [10]. The literature suggests using qualitative methods of testing over quantitative methods because qualitative methods generally result in a deeper understanding of user experience and gaps in the software [5,10]. Mixed methods were used in several studies [5,7,8,10], where quantitative methods (eg, questionnaires) were used to gain initial feedback followed by in-depth qualitative data collection (eg, interview or focus group) to ensure that the feedback was valuable and instructive. Using mixed methods allowed development teams to discover latent information and gain a better understanding of their participants’ experiences and needs [7,10].

When selecting a method of testing, development teams should particularly consider their specific testing sample. Methods of testing that may be effective for alpha testers may be unsuccessful when used with beta testers. Factors affecting the accuracy of testing data include the length of testing, question clarity and interpretation, question sequence, and the context in which testing takes place [15]. The general approach to software testing culminated in the collection and analysis of data, which were then used to create an iteration of the eHealth intervention.

Throughout the testing process of eHealth software, it is essential to uphold ethical principles due to the sensitive nature of health data and the potential impact on patient well-being. This includes ensuring patient privacy and confidentiality in compliance with national health care regulations, obtaining informed consent prior to participation, and adopting data protection and security measures to protect sensitive information [16,17].

## An Iterative Strategy

The success of eHealth development depends on the ability to adapt to the rapidly evolving nature of the digital world and changing user needs. Development teams interested in building eHealth interventions must consider how to ensure that testing occurs quickly, while still guaranteeing rigor and protecting privacy. Wilson et al [11] proposed an “mHealth Agile and User-Centered Research and Development Lifecycle” that combines an agile approach with traditional clinical trial phases to create high-quality mHealth interventions. Using an agile approach involves continuous iterative cycles that prioritize providing feedback on the software at frequent intervals. Compared to a linear approach to development, continuous evaluation of the developing software enhanced team collaboration, improved the quality of the software, and allowed for ongoing improvement with emphasis on end-user feedback [11].

Wilson et al [11] further suggested that alpha testing can be used to gain first-impression, surface-level insights from the development team, focusing on usability, desire to use, fulfillment of intended purpose, and safety. External testers can then be involved in beta testing, where usefulness, feasibility, and acceptability are evaluated [11]. When developing software for health care settings, several iterative rounds of alpha testing before proceeding to iterative rounds of beta testing may be most effective [11]. To further expand the breadth of feedback and reduce recall bias, iterative rounds of beta testing may be conducted with different samples of the end-user population. Depending on the revisions to the eHealth software after beta testing, a development team may decide to return to alpha testing to begin the process again. This highlights a within-and-between iterative strategy when conducting alpha and beta testing, offering the development team the flexibility to make continuous improvements to their eHealth software.

## Conclusion

Currently, there is great variation in how software testing is conducted in health care settings. However, we found that an iterative approach to testing is compatible with the need for an agile development technique for eHealth software. Qualitative methods of testing tend to yield more in-depth user experience feedback during beta testing and researchers benefit when two or more methods are used throughout the beta iteration. Yet, it remains unclear what prompts the transition from alpha to beta testing and when a repeat of testing is required. In the rapidly developing field of eHealth and mHealth interventions, it would be useful to have an agreed upon definition of what constitutes alpha and beta testing. Additionally, with the various frameworks of testing available, the best approach to software testing in health care settings remains unclear. To create clarity in this process, we suggest conducting a systematic review to understand and appraise the full scope of software testing practices within health care settings.

## Appendix

Multimedia Appendix 1 Characteristics of included studies.

## Abbreviations

mHealth: mobile health
NICU: neonatal intensive care unit
