# Supplementary material for: Software Testing of eHealth Interventions: Existing Practices and the Future of an Iterative Strategy
Source: JMIR Nurs. 2024 Jul 19;7:e56585. doi: 10.2196/56585 (PMC11297368; doi:10.2196/56585)
Supplement: Multimedia Appendix 1 [file nursing_v7i1e56585_app1.docx]

| Characteristics of Included Studies | | | | | | |
| --- | --- | --- | --- | --- | --- | --- |
| **Citation; Country** | **Purpose** | **Article Type** | **Alpha/Beta/** | **Product tested** | **Testing population** | **Methods of testing** |
| Ahonen et al [5]; USA | To test the development of eLearning modules for HFTAT program development | Primary Research | Alpha | eLearning software | Program directors (n = 5), staff members (n = 5) | User log, online questionnaire, focus group |
| Cho et al [7]; USA | To develop a theoretical approach and framework for the development of an HIV symptom self-management application | Primary Research | Both | Mobile app | Level 2: Persons living with HIV (PLWH) (n = 20)  Level 3: PLWH (n = 76) | Level 2: Eye tracking, think-aloud technique, task performance  Level 3: Health-IT Usability Evaluation Scale survey, interview |
| Fishbein et al [8]; USA | To develop and evaluate the usability and acceptability of a chemotherapy adherence and management application | Primary Research | Both | Mobile app | Alpha testing: research team (n = 4)  Beta testing: patients (n = 5) & oncology clinicians (n = 5) | Alpha testing: interview, feedback after review  Beta testing: follow-up assessment, interview |
| Hoffimann et al [9]; Portugal | To describe the development and testing of an audit application that assessed public open spaces (POS) quality | Primary Research | Both | Mobile app | Alpha testing: paper authors (n = 2) Beta testing: laypeople (n = 3) & external researchers (n = 6) | Alpha testing: qualitative feedback after use Beta testing: written qualitative feedback |
| Maramba et al [10]; United Kingdom | To describe the methods of usability testing used in eHealth applications | Review | Beta | eHealth software & applications | Most applications are tested by “real users” (e.g., HCP, caregivers, patients). Quantitative methods had higher average number of testers than qualitative testing. | Methods of testing included: quantitative (questionnaires, task completion) and qualitative ("Think-Aloud" protocol, interviews, and focus groups) and heuristic testing |
| Athilingamet al [6]; USA | To develop an application for heart failure patient education | Primary Research | Alpha | Mobile app | Alpha testing: patients with history of heart failure (n = 6)  Beta testing: patients at cardiology outpatient (n = 10) | Alpha testing: method not described  Beta testing: interview, questionnaire |
| Wilson et al [11]; Canada | To describe an mHealth research model which combines traditional clinical research methods and agile development methods | Framework | Both | eHealth software and applications | Alpha testing: project and engineering team members  Beta testing: external end-user sample | Alpha testing: evaluate the functionality of the product  Beta testing: determine whether the prototype meets the needs of the users  Clinical trial evaluation: ensure the product solves for which it was originally designed |
